# The Effects of Co-Culture of Embryonic Stem Cells with Neural Stem Cells on Differentiation

**DOI:** 10.3390/cimb44120416

**Published:** 2022-12-05

**Authors:** Ye Rim Kim, Si Won Jang, Jae Ho Han, Ga Rim Na, Hoon Jang, Hyun Woo Choi

**Affiliations:** 1 Department of Animal Science, Jeonbuk National University, Jeonju 54896, Republic of Korea; 2 Department of Agricultural Convergence Technology, Jeonbuk National University, Jeonju 54896, Republic of Korea; 3 Department of Life Science, Jeonbuk National University, Jeonju 54896, Republic of Korea

**Keywords:** embryonic stem cells, neural stem cells, cell–cell interaction, neural progenitors, neuronal differentiation

## Abstract

Researching the technology for in vitro differentiation of embryonic stem cells (ESCs) into neural lineages is very important in developmental biology, regenerative medicine, and cell therapy. Thus, studies on in vitro differentiation of ESCs into neural lineages by co-culture are expected to improve our understanding of this process. A co-culture system has long been used to study interactions between cell populations, improve culture efficiency, and establish synthetic interactions between populations. In this study, we investigated the effect of a co-culture of ESCs with neural stem cells (NSCs) in two-dimensional (2D) or three-dimensional (3D) culture conditions. Furthermore, we examined the effect of an NSC-derived conditioned medium (CM) on ESC differentiation. OG2-ESCs lost the specific morphology of colonies and Oct4-GFP when co-cultured with NSC. Additionally, real-time PCR analysis showed that ESCs co-cultured with NSCs expressed higher levels of ectoderm markers Pax6 and *Sox1* under both co-culture conditions. However, the differentiation efficiency of CM was lower than that of the non-conditioned medium. Collectively, our results show that co-culture with NSCs promotes the differentiation of ESCs into the ectoderm.

## 1. Introduction

Embryonic stem cells (ESCs), derived from the inner cell mass (ICM) of blastocysts, can proliferate indefinitely and differentiate into all cell lineages, including the germ line [1,2,3]. Because of the growing interest in the utilization of ESC-derived neural lineage cells for cell and tissue replacement therapies, a deep understanding of the mechanisms governing ESC differentiation into neural lineages is of great importance.

The fate of stem cells is controlled by various factors in the cellular microenvironment. These factors include cytokines, hormones, ionic gradients, extracellular matrix (ECM), cell substrates, and physical stimuli and ultimately govern whether a cell divides, differentiates, or dies by engaging in a multitude of intracellular signaling pathways [4,5,6,7]. In early mouse embryo gastrulation, pluripotent stem cells are separated into three germ layers: the ectoderm, mesoderm, and endoderm. High levels of activin/nodal signaling induce endoderm formation in mouse ESC cultures [8,9]. Early-stage induction of the mesoderm is regulated by Fetal Liver Kinase 1 (Flk-1) and Platelet-Derived Growth Factor Receptor (PDGFR) [10,11] and is correlated with a combination of Wnt, activin/nodule, and BMP signaling pathways [12,13]. Induction of the neuroectoderm depends on Fibroblast Growth Factor (FGF) signals endogenously produced by differentiating ESCs [14]; Bone Morphogenetic Protein (BMP), Wingless and Int-1 (Wnt), and activin/nodal signaling inhibiting the induction of the neuroectoderm [8,14,15]. The ectoderm develops into the surface ectoderm, the neural crest, and the neural tube; the surface ectoderm into the epidermis, hair, nails, and lens of the eye; the neural crest into the peripheral nervous system, adrenal medulla, and melanocytes; and the neural tube into the brain, spinal cord, and motor neurons [16].

Co-culture systems, which constitute more than two different types of cells in one culture dish, have been used to study the interaction between cell populations to improve culture efficiency or confirm interactions between populations. Co-culture methods can be divided into two main categories: direct and indirect. Direct co-culture methods allow cell–cell interactions between different types of cells, and this is usually achieved by controlling the location of adherent cells within the culture dish. However, in indirect methods, cells are physically separated by hanging cell culture inserts or overflow culture chambers, which allow communication only through secreted factors. Ou et al. used an indirect co-culture system to study intercellular communication between mouse ESCs and mouse epidermal keratinocytes or neonatal cardiomyocytes on a hanging cell culture insert [17]. Bahmani et al. co-cultured mouse adipose tissue-derived stem cells (ADSCs) with mouse ESCs to differentiate ADSCs into a neural lineage on a hanging cell culture insert [18]. In contrast, Rangappa et al. compared culturing in conditioned medium (CM) and direct co-culture of human mesenchymal stem cells (MSCs) with cardiomyocytes [4]. Bidarra et al. directly co-cultured human mesenchymal stem cells with endothelial cells [19], and Van der meer et al. directly co-cultured human endothelial cells with embryonic stem cell-derived pericytes [20]. The CM method does not allow direct cell-to-cell contact but enables exposure to soluble factors that are secreted from the target cells. Therefore, the CM method allows the isolation of the effects of chemical stimuli from physical stimuli. Kaplan et al. used the CM method to examine the effect of CM collected from crude testicular cell cultures on the differentiation of mouse ESCs into germ cell precursor cells and putative gametes [21]. However, CM is used for maintaining the undifferentiated state of the cells. Tsai et al. maintained the undifferentiated state of human ESCs using CM collected from autogeneic feeder cells [22]. 

We hypothesized that cell–cell interactions between ESCs and neural stem cells (NSCs) would affect ESC differentiation. Embryonic stem cells were cultured with NSCs (co-culture) or in the presence of CM containing secreted factors from NSCs. However, studies comparing the co-culture system and the CM culture system have not been reported for ESC differentiation into the neuroectoderm lineage. In this study, we found that two- and three-dimensional (2D and 3D, respectively) co-cultures of ESCs with NSCs contributed to the differentiation of ESCs into the ectoderm. The levels of the ectoderm markers *Sox1* and *Pax6* were significantly high when the NSC co-culture method was used. However, CM was not sufficient to stimulate stem cell differentiation. In summary, we suggest that co-culture with NSCs is effective for ESC differentiation into the neuroectoderm lineage on the basis of our observation that soluble factors alone are not sufficient to induce ESC differentiation and that physical contact with NSCs is required during ESC differentiation.

## 2. Materials and Methods

### 2.1. Types of Culture Medium

Four types of culture medium were used in this experiment. 

2i+leukemia inhibitory factor (LIF) medium: 1:1 mixture of DMEM/F12 (Gibco, Grand Island, NY, USA) and Neurobasal Medium (Gibco) containing 1× N2 supplement (Gibco), 2× B27 supplement (Gibco), 1× Penicillin/Streptomycin/Glutamine (Gibco), 50 µM β-mercaptoethanol (Gibco), 1000 U/mL LIF (Sigma-Aldrich, Chemical Co., St. Louis, MO, USA), 2i 1 µM PD0325901 (MEK inhibitor, Stemgent, Cambridge, MA, USA), and 3 µM CHIR99021 (GSK-3 inhibitor, Stemgent).

Fetal bovine serum (FBS) medium: DMEM (Gibco) containing 15% FBS (Gibco), 1× Penicillin/Streptomycin/Glutamine (Gibco), 1× MEM Non-Essential Amino Acids (Gibco), and 55 µM β-mercaptoethanol (Gibco).

FBS+LIF medium: FBS medium containing 1000 U/mL LIF (Sigma-Aldrich).

NSC medium: DMEM/F12 (Gibco) containing 1× N2 supplement (Gibco), 1× Penicillin/Streptomycin/Glutamine (Gibco), 50 µg/mL BSA (Sigma), 10 ng/mL EGF (R&D Systems, Minneapolis, MN, USA), and 10 ng/mL bFGF (Sigma).

### 2.2. Undifferentiated ESC Culture

Mouse ESC lines (OG2-ESC, E14, and E14-EGFP) were cultured without feeder cells in 2i+LIF medium (ESC culture medium). PCXLE-EGFP vector was applied for generating mouse transgenic ESCs expressing enhanced green fluorescent protein (EGFP). Transformed ESCs (E14-EGFP) expressed GFP even after several passages. The cells were incubated at 37 °C in a humidified atmosphere containing 5% CO_2_. The cells were passaged every 2 days.

### 2.3. Neural Stem Cell Culture

Mouse NSCs were obtained from *OG2^+/−^/ROSA26^+/−^* double-transgenic mice carrying *Oct4*-GFP and neo/lacZ. NSCs were cultured in common NSC culture medium (NSC medium) at 37 °C in a humidified atmosphere containing 5% CO_2_. The medium was changed on the second day. The cells were passaged every 3–4 days (80% confluence).

### 2.4. Mouse Embryonic Fibroblast Culture

Primary mouse embryo fibroblast (C3H-MEF) cell line was isolated from C3H mice on embryonic day (E) 12.5–14.5. The MEFs were passaged 4 times and maintained in DMEM supplemented with 15% FBS (FBS medium). The cells were incubated at 37 °C in a humidified atmosphere containing 5% CO_2_. The medium was changed on the second day. The cells were passaged every 4 days (at 100% confluence).

### 2.5. 2D Co-Culture of ESCs with NSC or MEF Feeders

NSCs were seeded in fibronectin (Sigma), and passage 4 MEFs were seeded in gelatin (Sigma)-coated 35 mm dishes. After 3 days, 10 µg/mL mitomycin C (MMC; Sigma) was added to produce the NSC and MEF feeders. The day after MMC treatment, ESCs were seeded and differentiated in FBS medium, FBS+LIF medium, and NSC medium. The cells were incubated at 37 °C in a humidified atmosphere containing 5% CO_2_ for 3 days. 

### 2.6. 3D Co-Culture with NSCs or MEFs

E14-EGFP or OG2-ESC (1.2 × 10^6^ cells/mL) were aggregated with NSC, MEF, or OG2-ESC (1.2 × 10^6^ cells/mL) in StemFIT 3D (Microfit, Seongnam, Korea) for 2 days to form embryoid bodies (EBs) in 2i+LIF medium. The EBs were then transferred to a suspension dish and cultured in differentiation medium for 6 days. The media used for differentiation were FBS, FBS+LIF, and NSC. The medium was changed every day. The cells were incubated at 37 °C in a humidified atmosphere containing 5% CO_2_. 

### 2.7. 2D or 3D Culture of ESCs in CM

NSCs (4 × 10^4^ cells/mL) were seeded in fibronectin, and passage 4 MEFs were seeded in gelatin-coated 60 mm dishes. After 3 days, 10 µg/mL mitomycin C was added to inactivate the cells. The day after MMC treatment, the cells were treated with 5 mL of FBS medium, FBS+LIF medium, and NSC medium and incubated at 37 °C in a humidified atmosphere containing 5% CO_2_ for 3 days. The CM was filtered with a 0.22 µM syringe filter immediately after collection. Because the CM does not contain cytokines, the corresponding cytokines were added to each CM before use. For 2D culture, ESCs were seeded in gelatin-coated 35 mm dishes. After 1 day, cells were treated with the pre-made CM and incubated at 37 °C in a humidified atmosphere of 5% CO_2_ for 6 days. The medium was changed every 2 days. In 3D culture, ESCs (1.2 × 10^6^ cells/mL) were seeded in StemFIT 3D (Microfit) for 2 days with standard ESC culture medium 2i+LIF to form uniformly sized EBs for differentiation. The EBs were then transferred to a suspension dish, cultured with pre-made CM, and incubated at 37 °C in a humidified atmosphere containing 5% CO_2_ for 6 days. The medium was changed every 2 days.

### 2.8. Reverse Transcription Polymerase Chain Reaction (qPCR)

Co-cultured E14-EGFPs were harvested and sorted using FACS Aria. Semi-quantitative reverse transcription (RT-PCR) for *Oct4, Nanog, Sox2, Pax6, Sox1, Mixl1, T, Gata4, Sox17, Otx2, Nestin, and Gapdh* was performed using standard procedures. Total RNA was extracted using the RNeasy Mini Kit (Qiagen, Valencia, CA, USA), and 1 µg of total RNA was reverse-transcribed with an Accupower CycleScript RT Premix (Bioneer, Seoul, Korea) according to the manufacturer’s instructions. cDNA was subjected to qPCR using Powerup SYBR Green master mix (Applied Biosystems, CA, USA). The primer sequences are listed in Table 1.

### 2.9. FACS Analysis and Sorting

The samples were dissociated into single cells by treatment with 0.25% trypsin-EDTA (Gibco). The cell pellets were then resuspended in PBS (Gibco) supplemented with 1% FBS (Gibco), 1 mM EDTA (Gibco), and 25 mM HEPES (pH 7.0, Gibco). The samples were analyzed and sorted using a FACS Aria III (Becton Dickinson) installed at the Center for University Research Facility (CURF) at Jeonbuk National University.

### 2.10. Equipment

The phase and fluorescence (GFP) images were captured using a Leica DMi8 microscope (Leica, Wetzlar, Germany). Scale bars of the images are indicated in the Figure legend of the Section 3.

### 2.11. Statistical Analysis

All of the experiments were performed three times, and the data of all repetitions of each experiment were collated and expressed as means ± standard error (SE) of the mean. Statistical tests were conducted using SAS version 9.4 (SAS Institute Inc., Cary, NC, USA), and a *p*-value < 0.05 was regarded as significant.

## 3. Results

### 3.1. NSC Feeder Induced Differentiation of ESCs

**Figure 1:**
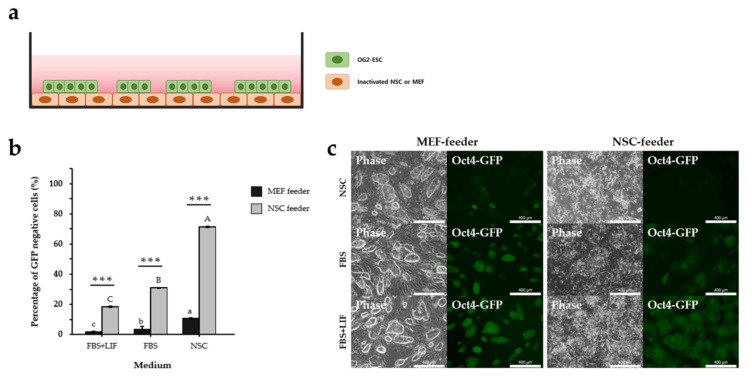
OG2-ESC co-cultured on neural stem cell (NSC)/mouse embryo fibroblast (MEF) feeder under 2D culture conditions. (**a**) Embryonic stem cells grown on inactivated NSC or MEF feeder. (**b**) Flow cytometry analysis of the percentage of Oct4-GFP-negative cells cultured on NSC or MEF feeders for 3 days. The asterisks represent significant differences in expression levels (Student’s *t*-test): *** *p* < 0.001. Uppercase and lowercase letters represent significant differences in the expression levels in various mediums (*p* < 0.001). Data are mean ± SE, *n* = 3 biological replicates. (**c**) Phase and fluorescence (GFP) images of OG2-ESCs cultured on NSC or MEF feeder for 3 days in fetal bovine serum (FBS), FBS + leukemia inhibitory factor (LIF), and NSC media. Scale bar = 400 µm.

For years, feeder cells have been used for promoting proliferation and self-renewal of ESCs through their extracellular secretions [1]. Various types of feeder cells, including MEF [23,24,25,26,27], MEF SNL line [28,29], human fetal and dermal fibroblasts [30,31,32], human foreskin fibroblasts [33], and murine amniocytes [34] have been used in human or mouse pluripotent cell cultures. However, according to a previous study, NSCs are not suitable as a feeder [35]. To investigate whether NSC-feeder cells promote ESC differentiation, we cultured ESCs on inactivated NSC- or MEF-feeder cells in a common ESC culture medium (FBS+LIF) (Figure 1a). We used the OG2-ESC cell line, which expresses GFP under the control of the *Oct4* promoter and distal enhancer, to estimate the level of ESC differentiation. After inducing differentiation in each feeder group for three days, we confirmed that the GFP expression of OG2-ESCs rapidly decreased in the NSC-feeder group (18.4%) (Figure 1b). To assess the potential default fate of ESCs from co-culture, we used a common NSC culture medium and FBS with or without LIF, which inhibits cell fate specification [36]. The ESCs lost the specific morphology of colonies and fluorescence in co-culture with NSC feeders and generally showed more differentiated patterns than MEF feeders in each medium (Figure 1c). In addition, because the GFP levels in OG2-ESC decreased under co-culture with the NSC feeders compared with that under co-culture with the MEF feeders (Figure 1c), we performed flow cytometry analysis to analyze the percentage of OG2-ESCs with GFP, which confirmed that GFP-negative cells increased significantly with NSC co-culture compared to that with MEF co-culture (Figure 1b). The percentage of differentiated ESCs was highest on NSC-feeder in NSC medium (62.8%). These results suggest that co-culture with NSC promotes ESC differentiation, and we inferred that this differentiation is more dramatic in the NSC medium than in the FBS and FBS+LIF medium.

### 3.2. NSC-CM Did Not Promote Differentiation of ESCs

**Figure 2:**
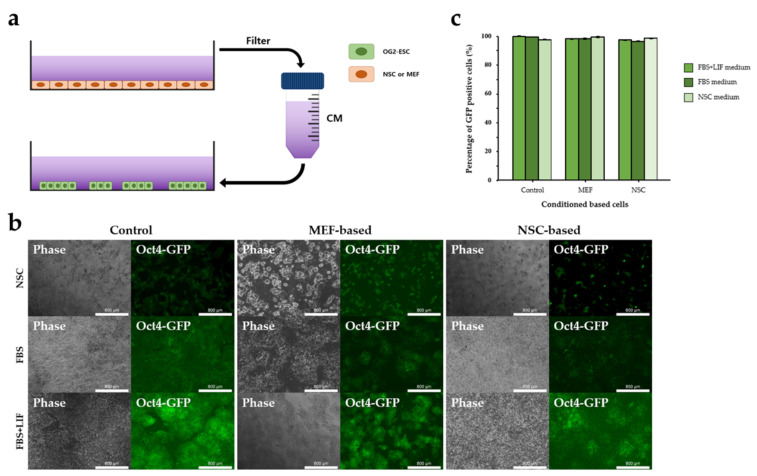
OG2-ESC cultured with CM under 2D condition. (**a**) CM prepared from the NSCs after 72 h in culture and used in the 2D culture of undifferentiated ESCs. (**b**) Phase and fluorescence (GFP) images of 2D OG2-ESCs cultured in non-conditioned medium or NSC- or MEF-based conditioned FBS, FBS+LIF, and NSC medium for 6 days. Scale bar = 800 µm. (**c**) Flow cytometry analysis of the percentage of Oct4-GFP-positive cells cultured in non-conditioned or CM under 2D condition for 6 days. Data are mean ± SE, *n* = 3 biological replicates.

**Figure 3:**
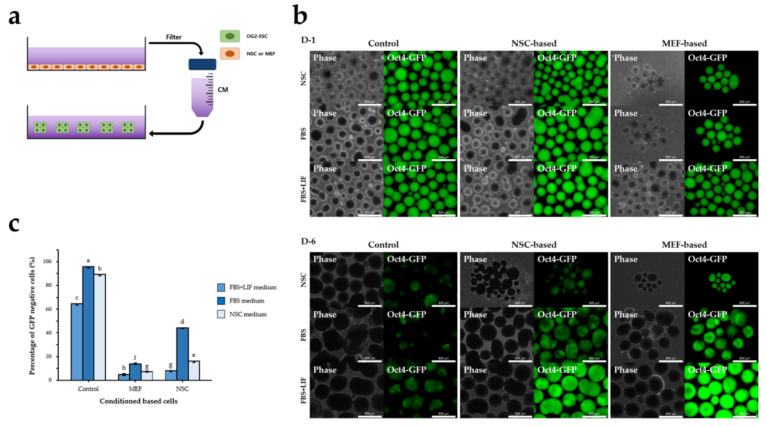
OG2-ESC cultured with CM under 3D condition. (**a**) CM prepared from the NSCs after 72 h in culture and used in the 3D culture of undifferentiated ESCs. (**b**) Day 1 and day 6 phase and fluorescence (GFP) images of 3D OG2-ESCs cultured in non-conditioned medium or NSC- or MEF-based conditioned FBS, FBS + LIF, and NSC medium for 6 days. Scale bar = 800 µm. (**c**) Flow cytometry analysis of the percentage of Oct4-GFP-negative cells cultured in non-conditioned or CM under 3D condition for 6 days. ^a–h^: Different lowercase letters represent significant differences between each condition (*p* < 0.001). Data are mean ± SE, *n* = 3 biological replicates.

We used the CM method to test whether direct physical cell–cell contact between NSCs and ESCs is essential for ESC differentiation. We cultured OG2-ESCs on gelatin-coated culture dishes without a feeder layer using pre-made CM (2D CM culture) (Figure 2a). After 3 days, most of the OG2-ESCs maintained GFP expression even after induction of differentiation using CM (Figure 2b), additionally, flow cytometry analysis confirmed that the percentage of GFP-negative cells did not differ between the control and CM groups (Figure 2c). Therefore, 3D culture was performed using the CM after inducing EB formation of OG2-ESC to promote differentiation of ESCs (Figure 3a). After being cultured for 2 days, the EBs were transferred to suspension dishes and differentiated. The EBs showed a tendency to lose GFP with time (Figure 3b). Flow cytometry analysis showed that the percentage of GFP-negative cells in the non-conditioned FBS medium was significantly higher than that in the CM. The other two types of CM (NSC and FBS+LIF) also showed the same trend as FBS CM (Figure 3c). These results suggested that CM was not effective in differentiating ESC, direct cell–cell attachment had a significant effect on cell differentiation, and this interaction is more important than paracrine signaling.

### 3.3. The Effect of 3D Co-Culture on Differentiation of ESCs

**Figure 4:**
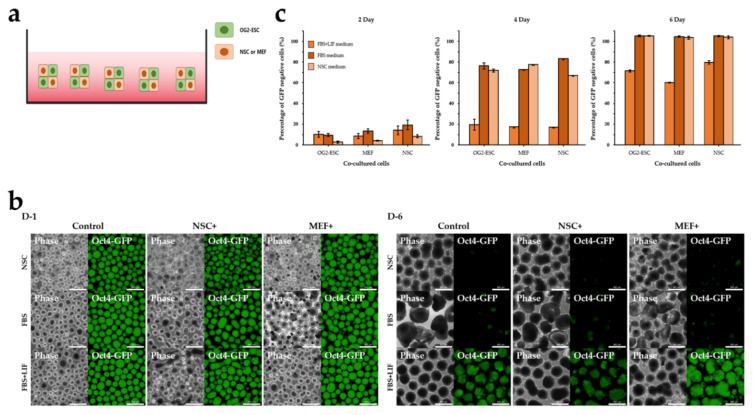
Co-culture of OG2-ESCs with NSC/MEF under 3D condition. (**a**) Embryonic stem cells aggregated with NSCs or MEFs and cultured on suspension dishes. (**b**) Day 1 and day 6 phase and fluorescence (GFP) images of 3D co-cultured OG2-ESCs on suspension dish in FBS, FBS+LIF, and NSC medium for 6 days. Scale bar = 800 µm. (**c**) Flow cytometry analysis of the percentage of Oct4-GFP-negative cells co-cultured with OG2-ESC, NSC, or MEF in FBS, FBS+LIF, and NSC media for 2, 4, and 6 days. Data are mean ± SE, *n* = 3 biological replicates.

To estimate the effect of EB on co-culture, ESCs were aggregated with NSCs or MEFs on StemFIT 3D dishes under 2i+LIF conditions (Figure 4a). Figure 4b shows the EBs photographed 1 and 6 days after co-culture. The boundary of the EB was rough and exhibited a tendency to lose GFP. On day 2, the percentage of GFP-negative cells was less than 20%; however, on day 4, it increased rapidly to 70% in MEF co-culture in FBS medium, 64% in NSC medium, 76% in NSC co-culture in FBS medium, and 56% in NSC medium. However, the FBS+LIF group still showed less than 15% GFP-negative cells. On day 6, in all experimental groups, ESCs lost more than 90% of GFP, except for those in the FBS+LIF medium (less than 60%) (Figure 4c). Thus, the differentiation rate varied according to the co-cultured cell type in the 2D cultures but not in the 3D cultures.

### 3.4. Neuroectodermal Differentiation of ESCs Was Improved by NSC Co-Culture

**Figure 5:**
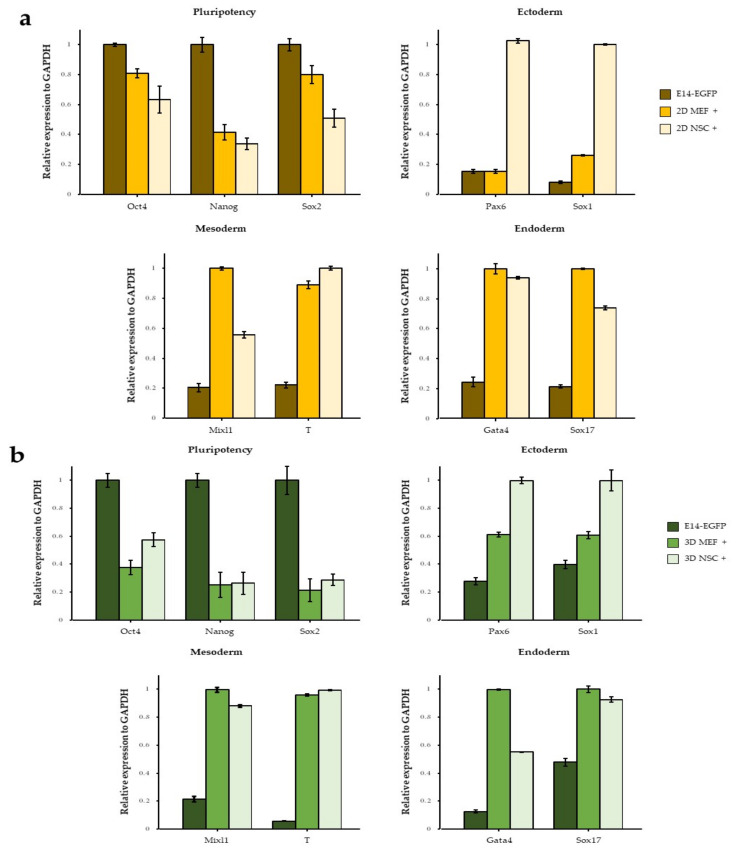
E14-EGFP co-cultured with NSC/MEF under 2D or 3D conditions express three embryonic germ markers. Pluripotency, ectoderm, mesoderm, and endoderm markers of E14-EGFPs 2D co-cultured with NSC or MEF feeder in FBS medium for 3 days (**a**) or 3D co-cultured with NSC or MEF in FBS medium for 6 days (**b**). EGFP-expressing cells were purified by FACS sorting. Data are mean ± SE, *n* = 3 biological replicates.

To further characterize ESCs co-cultured with NSCs, E14-EGFP was used for FACS sorting because it always expresses GFP, even after differentiation. We performed quantitative RT-PCR analysis of pluripotency and three germ layer markers in differentiated E14-EGFP cells on 2D or 3D co-culture with NSCs. The levels of the ESC markers *Oct4*, *Nanog*, and *Sox2* decreased after 2D co-culture (Figure 5a). The expression of neuroectoderm-specific markers, such as *Sox1* and *Pax6*, increased significantly in 2D NSC co-cultures. However, the mesoderm marker *Mixl1* and endoderm markers *Gata4* and *Sox17* were highly expressed in the 2D MEF co-culture. Interestingly, *T* was highly expressed in the NSC feeder. RT-PCR performed on 3D co-cultured ESCs showed the same tendency as that observed in 2D culture (Figure 5b). Since NSC medium was previously shown to promote the differentiation of ESCs in 2D NSC co-culture (Figure 1c), we additionally analyzed the expression of ectoderm markers. The expression levels of *Pax6* and *Sox1* were significantly higher in NSC medium than in MEF medium (Figure S1a). The real-time PCR results demonstrated that the culture environment caused significant differences in ectoderm marker expression. The expression of non-ectoderm genes (e.g., *Mixl1*) in NSC co-culture suggests that these specified NSC–ESC colonies are not completely committed to a neuroectoderm fate.

## 4. Discussion

ESCs can produce neural stem/progenitor cells under specific circumstances; however, generating pure and mature NSCs from ESCs remains challenging. Controlling and manipulating ESC differentiation culture conditions made it possible to generate an in vitro culture of neural lineage-specific cells from ESCs. In this study, we used an in vitro co-culture model to investigate ectodermal differentiation and improve its efficiency. We demonstrated that co-culture with NSCs induces the ESCs to differentiate into the ectoderm lineage. Under co-culture conditions, when ESCs directly contacted NSCs, the ectoderm-specific markers *Pax6* and *Sox1* of ESCs were upregulated. Although NSC-CM contained cytokines and other soluble elements secreted from the NSCs during normal growth, it was not sufficient to differentiate ESCs. We demonstrated that cell–cell contact between ESC and NSC is a cellular source of signals that can promote ESCs differentiation to the ectodermal lineage. 

We have shown that NSC co-culture induced the upregulation of genes involved in ectoderm formation, such as *Sox1* and *Pax6,* compared with MEF co-culture. In contrast, *Mixl1*, *Gata4*, *T*, and *Sox17* were activated in both co-cultures. We used the well-known ectoderm-specific markers *Pax6* and *Sox1* to estimate the ectodermal differentiation of ESCs. *Pax6* is a member of the murine-paired box gene family. During development, *Pax6* is expressed in the midbrain, forebrain, pituitary gland, olfactory epithelium, and eye [37,38]. Homozygous mutations of the *Pax6* gene in small eye mice were associated with severe central nervous system deformities, including the absence of the lenses and nasal cavities [39], multiple defects in early forebrain development [40], and sonic hedgehog (Shh)-dependent control of neuronal subtype specification [41]. *Sox1* encodes a transcription factor expressed in ectodermal cells. It contributes to the maintenance of neural progenitor cell identity and promotes neuronal lineage commitment [42]. *Sox1* appears to coincide with the induction of neuroectoderm, and it is expressed only in the neuroectoderm of mouse embryos [43]. Although mice with *Sox1* deficiency can survive, they exhibit lens defects and suffer from spontaneous seizures [44]. 

Direct physical cell–cell contact has been reported to play an important role in the differentiation of stem cells into various lineages or embryogenesis. Therefore, several studies have focused on the effects of adjacent cells in co-culture systems. For example, mouse EBs have been co-cultured with rat bone marrow stromal cells (BMSCs) for confirming the ability and efficiency of BMSCs to induce the differentiation of ESCs into cardiomyocytes. Compared with CM, direct co-culture was significantly effective in the differentiation of BMSCs into cardiomyocytes, but also in the CM from BMSCs. These results suggest that one or more soluble factors secreted from BMSCs play an important role in cardiac differentiation; however, direct cell–cell interactions are more important [45]. Rangappa et al. directly co-cultured human MSCs with cardiomyocytes to determine the phenotypical characteristics of cardiomyocytes and compared this approach with the CM method. The results demonstrated that soluble factors alone are not sufficient and that physical contact between cardiomyocytes and hMSCs is required to differentiate hMSCs into cardiomyocytes. Cell–cell interactions involve junctional complexes, including gap junctions, tight junctions, and desmosomes. Therefore, it is important that connexins [46], which are critical in the formation of gap junctions, are expressed in stem cells. Therefore, connexins may be involved in signaling signals that occur during co-culture experiments by opening cell-cell contacts and aiding in the transmission of intracellular signaling molecules. Cell–cell contact can also result in cell shape changes due to the mechanical stretch imposed by neighboring cells. In addition, the homology of cell surface receptors and other proteins related to cell–cell adhesion could activate differentiation-associated genes [4]. From this perspective, the upregulation of neuroectodermal markers in ESCs co-cultured with NSCs appears to be caused by gap junctional interactions and other proteins. 

In contrast, CM provides soluble factors that are secreted from independent cells without a direct connection. Therefore, the CM method allows the separation of the effects of chemical and physical stimuli. Kaplan et al. used the CM method to examine the effect of CM collected from crude testicular cell culture on the differentiation of mouse ESCs into germ cell precursor cells and putative gametes. Most growth factors required for the transformation of germ stem cells into differentiated gametes are present in the testes at developmental stages. When the EBs were cultured in CM prepared from crude testicular cell culture, they developed into ovarian structures containing putative oocytes. Although the cytokine content of the CM was not analyzed, this study suggests that growth factors secreted by testicular cells are responsible for the transformation of ESCs into gametes [21]. However, CM has also been used to maintain the undifferentiated state of cells. Tsai et al. maintained the undifferentiated state of human ESCs using CM collected from autogeneic feeder cells. In Matrigel feeder-free culture conditions, CM collected from MEF produced important effects on the cytoskeleton remodeling of human ESCs. The gene expression profiles of mRNAs, microRNAs, and proteins between the undifferentiated control ESC and CM-cultured ESCs were very similar. In addition, the undifferentiated state of cells was evidenced by high expression levels of pluripotency markers and low expression levels of differentiation markers. In summary, CM supported the undifferentiated growth of human ESCs [22].

Co-culturing and CM methods can also be applied to organoid studies. Organoids are 3D cell culture models that are grown from stem cells; they mimic the structural and functional characteristics of an organ [47]. Organoids can provide the opportunity to study the multi-lineage differentiation of the original tissue more closely than 2D cell culture systems. Typically, organoids can be developed from embryonic stem cells, induced pluripotent stem cells, and organ-specific adult stem cells [48]. Human induced pluripotent stem cells (iPSCs) and bone marrow-derived MSC co-culture were used for investigating the impact of 3D hybrid spheroids on in vitro dorsal cortical differentiation and secretion of extracellular matrices and trophic factors. Neural differentiation was promoted along with upregulation of neural markers [49]. Baghdadi et al. analyzed mouse intestinal organoid cultures with CM isolated from mucosal enteric glial cell (EGC) cultures and investigated the mechanisms of intestinal stem cell interaction with mucosal EGCs, which has been shown to enhance stem cell activity [50]. Differentiation of stem cells into a specific lineage is important for superior organoid formation [51]. Our experiments showed that 3D co-culture of ECS with NSC promoted the upregulation of neuroectodermal markers. Therefore, this method may be useful for the study of brain organoid model formation and establishment of neural lineage cell lines.

## 5. Conclusions

In summary, the current study investigated the effect of co-culture with NSCs on ESC differentiation. We found that OG2-ESCs lost the *Oct4*-GFP expression when co-cultured with NSC under 2D and 3D conditions. We also demonstrated that co-culture with NSC can promote the upregulation of neuroectodermal markers *Pax6* and *Sox1* of ESC. This method will be useful for the study of brain organoid model formation and establishment of neural lineage cell lines.

## Figures and Tables

**Table 1:** Gene-specific primer sequences for real-time reverse transcription polymerase chain reaction (RT-PCR).

Gene		Sequence 5′-3′
*Gapdh*	F	ATGAATACGGCTACAGCAACAGG
	R	CTCTTGCTCAGTGCCTTGCTG
*Oct4*	F	CCAATCAGCTTGGGCTAGAG
	R	CTGGGAAAGGTGTCCCTGTA
*Nanog*	F	CTTTCACCTATTAAGGTGCTTGC
	R	TGGCATCGGTTCATCATGGTA
*Sox2*	F	CATGAGAGCAAGTACTGGCAAG
	R	CCAACGATATCAACCTGCATGG
*Pax6*	F	ACCAGTGTCTACCAGCCAATCC
	R	GCACGAGTATGAGGAGGTCTGA
*Sox1*	F	GCCGAGTGGAAGGTCATGTC
	R	TTGAGCAGCGTCTTGGTCTTG
*Mixl1*	F	TCCTCCATTGCCCTGCTCCT
	R	ACGCCTCCTCCAGTCATGCT
*T*	F	ATCAGAGTCCTTTGCTAGGTAG
	R	GTTACAATCTTCTGGCTATGC
*Gata4*	F	CAGCAGCAGCAGTCAAGAGATG
	R	ACCAGGCTGTTCCAAGAGTCC
*Sox17*	F	TTCTGTACACTTTAATGAGGCTGTTC
	R	TTGTGGGAAGTGGGATCAAG

*Gapdh*, glyceraldehyde 3-phosphate dehydrogenase; *Oct4*, POU domain, class 5, transcription factor 1; *Nanog*, Nanog homeobox; *Sox2*, SRY-box transcription factor 2; *Pax6*, paired box 6; *Sox1*, SRY-box transcription factor 1; *Mixl1*, Mix paired-like homeobox; *T*, T-box transcription factor T; *Gata4*, GATA binding protein 4; *Sox17*, SRY-box transcription factor 17.

## Data Availability

The datasets generated during and/or analyzed during the current study are available from the corresponding author on reasonable request.

## Supplementary Materials

The following supporting information can be downloaded at https://www.mdpi.com/article/10.3390/cimb44120416/s1. Figure S1: E14-EGFP co-cultured with NSC under 2D condition express ectoderm markers *Pax6* and *Sox1*. (**a**) *Pax6* and *Sox1* expression level in E14-EGFPs 2D co-cultured with NSC feeder in NSC or FBS medium for 3 days. EGFP-expressing cells were purified by FACS sorting. Data are mean ± SE, *n* = 3 biological replicates.
