# Supplementary figures and images for: The Effects of Co-Culture of Embryonic Stem Cells with Neural Stem Cells on Differentiation
Source: Curr Issues Mol Biol. 2022 Dec 5;44(12):6104–16. doi: 10.3390/cimb44120416 (PMC9776753; doi:10.3390/cimb44120416)

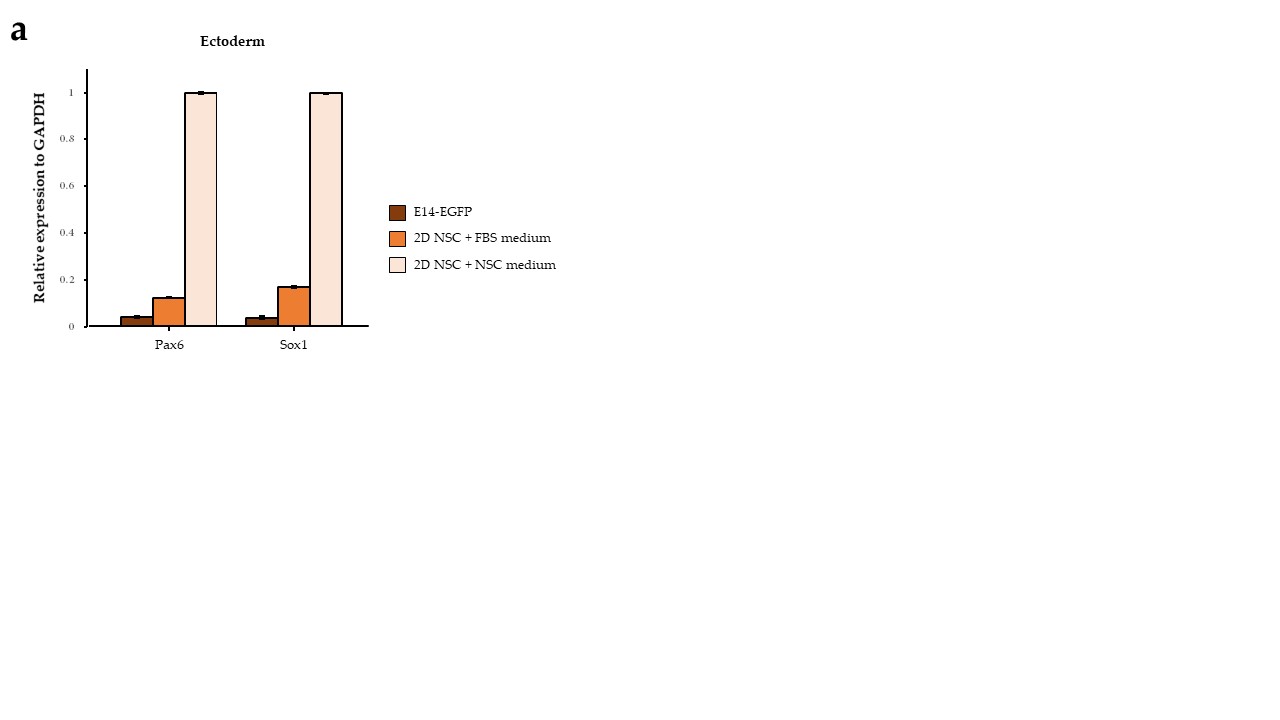

Supplement: Supplementary file 1 [file cimb-44-00416-s001.zip › Supplementary Materials_Figure S1.jpg]
